# Some weighted inequalities for Hausdorff operators and commutators

**DOI:** 10.1186/s13660-017-1588-4

**Published:** 2018-01-05

**Authors:** Amjad Hussain, Amna Ajaib

**Affiliations:** Department of Mathematics, Quaid-I-Azam University, 45320, Islamabad, 44000 Pakistan

**Keywords:** 26D15, 42B35, 42B25, 46E30, Hausdorff operators, commutators, weighted central Morrey spaces, central-*BMO* functions, sharp bounds

## Abstract

In this paper, we consider the problem of boundedness of Hausdorff operator on weighted central Morrey spaces. In particular, we obtain sharp bounds for Hausdorff operators on power weighted central Morrey spaces. Analogous results for the commutators of Hausdorff operators when the symbol functions belong to weighted central-*BMO* spaces are obtained as well.

## Introduction

In recent years, the Hausdorff operator has gained much attention. This is mainly because of seminal work done by Liflyand and Móricz in [1]. In the paper, they considered the one dimensional Hausdorff operator

$$h_{\Phi}(x)= \int_{0}^{\infty}\frac{\Phi(t)}{t}f\biggl( \frac{x}{t}\biggr)\,dt, $$

on real Hardy spaces. In [2], Lerner and Liflyand gave the following extension of $h_{\Phi}$ to Euclidean space $\mathbb {R}^{n}$ for $n\ge2$:

1.1 $$\begin{aligned} H_{\Phi,A}f(x)= \int_{\mathbb {R}^{n}} \frac {\Phi(y)}{ \vert y \vert ^{n}} f\bigl(A(y)x\bigr) \,dy, \end{aligned}$$

where $A(y)$ is an $n\times n$ matrix satisfying non-singularity conditions almost everywhere in the support of a fixed integrable function Φ. After the appearance of references [1, 2], it was natural to study, refine and extend the existing results on relevant function spaces. A number of significant studies have been undertaken in this regard like for example boundedness of one and multidimensional Hausdorff operators on Hardy, $L^{p}$ and *BMO* spaces [3–8]. Besides, many authors have contributed a lot towards obtaining new estimates on other function spaces. Among them we refer to [9–14] and the references therein.

On the other hand weighted norm inequalities for Hausdorff operators on function spaces have recently been reported in the literature, which includes boundedness of Hausdorff operator on power weighted Hardy spaces [15, 16], weighted Herz-type Hardy spaces [17] and on weighted Herz space on the Heisenberg group [18].

In harmonic analysis the study of commutator operators on function spaces is also considered to be very important. It is to be noted that the commutators generated by Hausdorff operators with other functions are not widely discussed in the past. In a survey of available literature one can find very few papers on this topic [19–23]. We define commutators $H_{\Phi,A}^{b}$ of $H_{\Phi,A}$ with locally integrable function *b* as

1.2 $$\begin{aligned} H_{\Phi,A}^{b}(f)=bH_{\Phi,A}(f)-H_{\Phi,A}(bf). \end{aligned}$$

When $A(y)=\operatorname{diag}[1/ \vert y \vert ,1/ \vert y \vert ,\ldots,1/ \vert y \vert ]$, we obtain the commutators $H_{\Phi}^{b}$ of $H_{\Phi}$, where $H_{\Phi}$ is given by

1.3 $$\begin{aligned} H_{\Phi}f(x)= \int_{\mathbb {R}^{n}} \frac {\Phi(y)}{ \vert y \vert ^{n}} f\biggl(\frac{x}{ \vert y \vert }\biggr) \,dy. \end{aligned}$$

In [20], the authors have obtained some size conditions on Φ such that the commutator generated by Hausdorff operator $H_{\Phi}$ with Lipschitz function *b* is bounded on classical Morrey spaces. However, the question of the boundedness of $H_{\Phi,A}^{b}$ on function spaces is yet to be answered.

The purpose of this study is twofold. Firstly, motivated by the work in [17, 18], we give estimates for the Hausdorff operator on weighted central Morrey space. In addition, under some assumption on $A(y)$, we work out the operator norm for $H_{\Phi,A}$ on power weighted central Morrey spaces. Secondly, we try to fill the gap to existing theory of the commutator of Hausdorff operators by defining a new type of commutators in (1.2) and establishing the weighted estimates for such commutator operators. More precisely, under some assumptions on $A(y)$, we give necessary and sufficient conditions on the function Φ such that $H_{\Phi,A}^{b}$ is bounded on power weighted central Morrey spaces.

This paper is organized as follows. In the next section, we will introduce some notations and definitions along with some necessary lemmas to be used in the subsequent sections of this paper. Our main results regarding boundedness of Hausdorff operators on weighted central Morrey spaces are stated and proved in the third section. Finally, the last section is devoted to obtaining weighted estimates for the commutators of Hausdorff operator.

## Notations and definitions

In 1938, Morrey [24] carried out a systematic study to investigate the local behavior of solutions to a certain kind of partial differential equations and introduced a new function space, what is called Morrey space. For $1\le q<\infty$ and $-1/q<\lambda<0$, the Morrey space $M^{q,\lambda}(\mathbb {R}^{n})$ was defined as the set of all locally integrable functions *f* satisfying

$$\Vert f \Vert _{M^{q,\lambda}(\mathbb {R}^{n})}=\sup_{a\in \mathbb {R}^{n}, R>0} \biggl( \frac{1}{ \vert B(a,R) \vert ^{1+\lambda q}} \int_{B(a,R)} \bigl\vert f(x) \bigr\vert ^{q}\,dx \biggr)^{1/q}< \infty, $$

where $B(a,R)$ is the Euclidean ball centered at *a* with radius *R* and $\vert B(a,R) \vert $ is its Lebesgue measure. If $B(a,R)$ is replaced by $B(0,R)$ in the above definition, then the function space is the central Morrey space $\dot{M}^{q,\lambda}(\mathbb {R}^{n})$ introduced in [25] with the norm condition

$$\Vert f \Vert _{\dot{M}^{q,\lambda}(\mathbb {R}^{n})}=\sup_{R>0} \biggl( \frac{1}{ \vert B(0,R) \vert ^{1+\lambda q}} \int_{B(0,R)} \bigl\vert f(x) \bigr\vert ^{q}\,dx \biggr)^{1/q}< \infty. $$

For the boundedness of the commutator operator on function spaces of central nature, one usually looks for a corresponding function class to which the symbol function *b* belongs and which has *BMO*-type behavior at the origin. Having such a property an appropriate function space is the homogeneous central mean oscillation space $C\dot{M}O^{q}(\mathbb {R}^{n})$. Let $f_{B(0,R)}$ denote the mean value of *f* over $B(0,R)$, then we say that any locally integrable function *f* is in $C\dot {M}O^{q}(\mathbb {R}^{n})$ if

$$\Vert f \Vert _{{C\dot{M}O}^{q}(\mathbb{R}^{n})}= \sup_{R>0} \biggl( \frac{1}{ \vert B(0,R) \vert } \int_{B(0,R)} \bigl\vert f(x)-f_{B(0,R)} \bigr\vert ^{q}\,dx \biggr)^{1/q}< \infty. $$

For a detailed study of $C\dot{M}O^{q}(\mathbb {R}^{n})$ space we refer the interested reader to [25, 26].

Obviously, $BMO(\mathbb {R}^{n})\subset C\dot{M}O^{q}(\mathbb {R}^{n})$ for $1\le q<\infty$. However, the two spaces differ in their properties. For example $C\dot{M}O^{q}(\mathbb {R}^{n})$ depends on *q* and $C\dot {M}O^{q}(\mathbb{R}^{n}) \subset C\dot{M}O^{p}(\mathbb{R}^{n})$, $1 \leq p < q< \infty$. Therefore, there is no analogy of the John-Nirenberg inequality of $BMO(\mathbb {R}^{n})$ for $C\dot {M}O^{q}(\mathbb {R}^{n})$. The space $BMO(\mathbb {R}^{n})$ is the mean oscillation function space satisfying the following norm condition:

$$\Vert f \Vert _{BMO(\mathbb{R}^{n})}=\sup_{B} \frac{1}{ \vert B \vert } \int_{B} \bigl\vert f(x)-f_{B} \bigr\vert \,dx. $$

Muckenhoupt [27] firstly introduced the theory of $A_{p}$ weights while studying Hardy-Littlewood maximal functions on weighted $L^{p}$ spaces. A weight *w* is a nonnegative, locally integrable function on $\mathbb {R}^{n}$. For a given subset *E* of $\mathbb {R}^{n}$, we denote by $w(E)$ the weighted measure of *E*, that is, $w(E)=\int_{E}w(x)\,dx$. Also, by $p^{\prime}$ we denote the conjugate index of *p*, satisfying $1/p+1/{p^{\prime}}=1$.

### Definition 2.1

A weight *w* is said to belong to the Muckenhoupt class $A_{p}$, $1< p<\infty$, if there exists a positive constant *C* such that, for every ball $B\subset\mathbb {R}^{n}$,

$$\begin{aligned} \biggl(\frac{1}{ \vert B \vert } \int _{B}w(x)\,dx \biggr) \biggl(\frac{1}{ \vert B \vert } \int _{B}w(x)^{-1/(p-1)}\,dx \biggr)^{p-1}\le C. \end{aligned}$$

Also, $w\in A_{1}$ if there exists a positive constant *C* such that, for every ball $B\subset\mathbb {R}^{n}$,

$$\biggl(\frac{1}{ \vert B \vert } \int_{B}w(x)\,dx \biggr)\le C\operatorname*{essinf}_{x\in B} w(x). $$

For $p=\infty$, we define $A_{\infty}=\bigcup_{1\le p<\infty}A_{p}$.

### Definition 2.2

([28])

A weight *w* is said to belong to the reverse Hölder class $RH_{r}$ if there exists a fixed positive constant *C* and $r>1$ such that, for every ball $B\subset\mathbb {R}^{n}$,

$$\begin{aligned} \biggl(\frac{1}{ \vert B \vert } \int _{B}w^{r}(x)\,dx \biggr)^{1/r}\le \frac{C}{ \vert B \vert } \int_{B}w(x)\,dx. \end{aligned}$$

It is also well known for $s > p$ that $A_{p}\subset A_{s}$ and that if $w\in A_{p}$, $1 < p < \infty$, then $w\in A_{q}$ for some $1 < q < p$. The infimum of all *q* such that $w\in A_{q}$ is denoted by $q_{w}$ and is known as the critical index for *w*. In addition, if $w\in RH_{r}$, $r>1$, then for some $\epsilon>0$ we have $w\in RH_{r+\epsilon}$. We therefore use $r_{w}\equiv\sup\{r>1:w\in RH_{r}\}$ to denote the critical index of *w* for the reverse Hölder condition.

A special class of Muckenhoupt $A_{p}$ weights is the power function $\vert x \vert ^{\alpha}$. It is well known that $\vert x \vert ^{\alpha}\in A_{1}$ if and only if $-n<\alpha \le0$. Also, for $0<\alpha<\infty$, $\vert x \vert ^{\alpha}\in\bigcap_{(n+\alpha)/n< p<\infty} A_{p}$, where $(n+\alpha)/n$ is known as the critical index of $\vert x \vert ^{\alpha}$.

Here we state some propositions regarding $A_{p}$ weights, which will be helpful in obtaining weighted estimates for Hausdorff operator and their commutators.

### Proposition 2.3

([28, 29])

*Let* $w\in A_{p}\cap RH_{r}$, $p\ge1$ *and* $r>1$. *Then there exist constant* $C_{1},C_{2}>0$ *such that*

$$C_{1} \biggl(\frac{ \vert E \vert }{ \vert B \vert } \biggr)^{p}\le \frac{w(E)}{w(B)}\le C_{2} \biggl(\frac{ \vert E \vert }{ \vert B \vert } \biggr)^{(r-1)/r}, $$

*for any measurable subset* *E* *of the ball* *B*. *In general*, *for any* $\lambda>1$,

$$w(B(x_{0},\lambda R)\le\lambda^{np}w\bigl(B(x_{0},R) \bigr). $$

### Proposition 2.4

([17])

*Let* *f* *be a nonnegative locally integrable function*. *If* $w\in A_{p}$, $p\ge1$, *then*

$$\frac{1}{ \vert B(x_{0},R) \vert } \int_{B(x_{0},R)}f(x)\,dx\le C \biggl(\frac{1}{w(B(x_{0},R))} \int_{B(x_{0},R)}f^{p}(x)w(x)\,dx \biggr)^{1/p}. $$

Let *w* be a weight function on $\mathbb {R}^{n}$, for any measurable set $E\subset\mathbb {R}^{n}$, the weighted Lebesgue space $L^{p}(E;w)$ is the space of all functions satisfying

$$\Vert f \Vert _{L^{p}(E;w)}= \biggl( \int_{E} \bigl\vert f(x) \bigr\vert ^{p}w(x)\,dx \biggr)^{1/p}< \infty. $$

In 2009, Komori and Shirai [30] introduced the weighted Morrey space and studied the properties of classical operators on this space. Here, we only give the definition of the weighted central Morrey space.

### Definition 2.5

Let $\lambda\in\mathbb {R}$, $1\le q<\infty$ and *w* is a weight function on $\mathbb {R}^{n}$. Then the weighted central Morrey space $\dot{M}^{q,\lambda}(\mathbb {R}^{n};w)$ is defined by

$$\dot{M}^{q,\lambda}\bigl(\mathbb {R}^{n};w\bigr)= \bigl\{ f \in L_{\mathrm{ loc}}^{q}\bigl(\mathbb{R}^{n};w\bigr): \Vert f \Vert _{\dot {M}^{q,\lambda}(\mathbb {R}^{n};w)}< \infty \bigr\} , $$

where

$$\Vert f \Vert _{\dot{M}^{q,\lambda}(\mathbb {R}^{n};w)}=\sup_{R>0} \biggl( \frac{1}{w(B(0,R))^{1+\lambda q}} \int_{B(0,R)} \bigl\vert f(x) \bigr\vert ^{q}w(x)\,dx \biggr)^{1/q}< \infty. $$

If *w* is a weight function and $f_{B,w}=\frac{1}{w(B(0,R))}\int _{B(0,R)} \vert f(y) \vert w(y)\,dy$, then the weighted central mean oscillation space can be defined as follows.

### Definition 2.6

Let $1< q<\infty$, and *w* be a weight function. Then we say a function $f \in L_{\mathrm{loc}}^{q}(\mathbb{R}^{n};w)$ belongs to the weighted central mean oscillation space $C\dot {M}O^{q}(\mathbb{R}^{n};w)$ if

$$\Vert f \Vert _{C\dot{M}O^{q}(\mathbb{R}^{n};w)}=\sup_{R>0} \biggl( \frac{1}{w(B(0,R))} \int_{B(0,R)} \bigl\vert f(x)-f_{B,w} \bigr\vert ^{q}w(x)\,dx \biggr)^{1/q}< \infty. $$

In the sequel, we shall use the notation $A \preceq B$, meaning that there exists a constant $C>0$ independent of all essential values and variables such that $A \le C B$. We shall use the notation $A\simeq B$, meaning that there exist constants $C>0$ and $c>0$ independent of all essential values and variables such that $cB\le A\le CB$. Moreover, we will denote a weight from the Muckenhoupt $A_{p}$ class by *w*. However, when the weight is reduced to the power function, we will denote it by *v*, that is, $v(x)= \vert x \vert ^{\alpha}$.

Finally, for an invertible matrix *D* we will use the norm

2.1 $$\begin{aligned} \Vert D \Vert =\sup_{x\in\mathbb {R}^{n},x\neq0}\frac{ \vert Dx \vert }{ \vert x \vert }. \end{aligned}$$

Then it is easy to see that

2.2 $$\begin{aligned} \begin{aligned} \Vert D \Vert ^{-n}\le \bigl\vert \det\bigl(D^{-1}\bigr) \bigr\vert \le \bigl\Vert D^{-1} \bigr\Vert ^{n}. \end{aligned} \end{aligned}$$

### Proposition 2.7

*Let* *α* *be a real number*, *D* *is any nonsingular matrix and* $x\in\mathbb {R}^{n}$, *then we have the following results*:

(i) $$\begin{aligned} \begin{aligned} v(D x)&\preceq \textstyle\begin{cases} \Vert D \Vert ^{\alpha}v(x) & \textit{if } \alpha>0,\\ \Vert D^{-1} \Vert ^{-\alpha} v(x) & \textit{if } \alpha \le0; \end{cases}\displaystyle \end{aligned} \end{aligned}$$
(ii) $$\begin{aligned} v\bigl(B\bigl(0, \Vert D \Vert R\bigr)\bigr)= \Vert D \Vert ^{n+\alpha} v\bigl(B(0,R)\bigr). \end{aligned}$$

### Proof

The proof follows from the definition of $v(x)$ and (2.1). □

Henceforth, for the sake of convenience, we will denote $B(0,R)$ by *B*.

## Bounds for $H_{\Phi,A}$ on weighted central Morrey space

This section is devoted to stating and proving results on the boundedness of $H_{\Phi,A}$ on a weighted central Morrey space.

### Main results

We now present the main results for this section.

#### Theorem 3.1

*Let* $1\le q_{1},q_{2}<\infty$, $\lambda<0$. *Suppose that* $w\in A_{1}$ *with the critical index* $r_{w}$ *for the reverse Hölder condition and suppose that* $q_{1}>q_{2}r_{w}/(r_{w}-1)$.

*Then for any* $1<\delta<r_{w}$

$$\begin{aligned} \begin{aligned} \Vert H_{\Phi,A}f \Vert _{\dot{M}^{q_{2},\lambda}(\mathbb {R}^{n};w)}& \preceq K_{1} \Vert f \Vert _{\dot{M}^{q_{1},\lambda }(\mathbb {R}^{n},w)}, \end{aligned} \end{aligned}$$

*where*

$$\begin{aligned} \begin{aligned} K_{1}&= \int_{ \Vert A(y) \Vert < 1}\frac{ \vert \Phi (y) \vert }{ \vert y \vert ^{n}} \bigl\vert \det A^{-1}(y) \bigr\vert ^{1/q_{1}} \bigl\Vert A(y) \bigr\Vert ^{n\lambda +n/q_{1}}\,dy \\ &\quad{} + \int_{ \Vert A(y) \Vert \ge1}\frac{ \vert \Phi (y) \vert }{ \vert y \vert ^{n}} \bigl\vert \det A^{-1}(y) \bigr\vert ^{1/q_{1}} \bigl\Vert A(y) \bigr\Vert ^{n/q_{1}+n\lambda(\delta-1)/\delta}\,dy. \end{aligned} \end{aligned}$$

In the case that general weights are replaced by a power function, the result can be stated in the form of the following theorem.

#### Theorem 3.2

*Let* $1\le q<\infty$, $-1/q\le\lambda<0$.

(i) *If* $0<\alpha<\infty$,
  $$\begin{aligned} \begin{aligned} \Vert H_{\Phi,A}f \Vert _{\dot{M}^{q,\lambda}(\mathbb {R}^{n};v)}& \preceq K_{2} \Vert f \Vert _{\dot{M}^{q,\lambda }(\mathbb {R}^{n},v)}, \end{aligned} \end{aligned}$$
  *where*
  $$\begin{aligned} \begin{aligned} K_{2}&= \int_{\mathbb {R}^{n}}\frac{\Phi(y)}{ \vert y \vert ^{n}} \bigl\vert \det A^{-1}(y) \bigr\vert ^{1/q} \bigl\Vert A(y) \bigr\Vert ^{(n+\alpha)(\lambda+1/q)} \bigl\Vert A^{-1}(y) \bigr\Vert ^{\alpha/q}\,dy . \end{aligned} \end{aligned}$$
(ii) *If* $-n<\alpha\le0$, *then*
  $$\begin{aligned} \begin{aligned} \Vert H_{\Phi,A}f \Vert _{\dot{M}^{q,\lambda}(\mathbb {R}^{n};v)}& \preceq K_{3} \Vert f \Vert _{\dot{M}^{q,\lambda }(\mathbb {R}^{n},v)}, \end{aligned} \end{aligned}$$
  *where*
  $$\begin{aligned} \begin{aligned} K_{3}&= \int_{\mathbb {R}^{n}}\frac{\Phi(y)}{ \vert y \vert ^{n}} \bigl\vert \det A^{-1}(y) \bigr\vert ^{1/q} \bigl\Vert A(y) \bigr\Vert ^{n(\lambda+1/q)+\alpha\lambda}\,dy. \end{aligned} \end{aligned}$$

Especially, if $\Vert A^{-1}(y) \Vert $ and $\Vert A(y) \Vert ^{-1}$ are comparable, we obtain the following sharp result.

#### Theorem 3.3

*Let* $1\le q<\infty$, $-1/q\le\lambda<0$, $-n<\alpha<\infty$, *and* Φ *be a nonnegative function*. *Suppose that there is a constant* *C* *independent of* *y* *such that* $\Vert A^{-1}(y) \Vert \le C \Vert A(y) \Vert ^{-1}$ *for all* $y\in\operatorname{supp}(\Phi)$, *then* $H_{\Phi,A}$ *is bounded on* $\dot{M}^{q,\lambda}(\mathbb {R}^{n};v)$ *if and only if*

$$\begin{aligned} \begin{aligned}K_{4}= \int_{\mathbb {R}^{n}}\frac{\Phi(y)}{ \vert y \vert ^{n}}\vert \bigl\Vert A(y) \bigr\Vert ^{(n+\alpha)\lambda}\,dy< \infty. \end{aligned} \end{aligned}$$

#### Remark 3.4

Let $A(y)=\operatorname{diag}[1/\mu_{1}(y),\ldots,1/\mu_{n}(y)]$ for $\mu _{i}(y)\neq0$ ($i=1,2,\ldots,n$). Define

$$m(y)=\min\bigl\{ \bigl\vert \mu_{1}(y) \bigr\vert ,\ldots, \bigl\vert \mu _{n}(y) \bigr\vert \bigr\} ,\qquad M(y)=\max\bigl\{ \bigl\vert \mu_{1}(y) \bigr\vert ,\ldots, \bigl\vert \mu_{n}(y) \bigr\vert \bigr\} . $$

For a constant $C\ge1$ independent of *y* if $M(y)\le Cm(y)$, then it can easily be verified that $A(y)$ satisfies the assumptions of Theorem 3.3.

In the remaining of this section we will prove Theorems 3.1-3.3.

### Proof of Theorem 3.1

For a fixed ball $B\subset\mathbb {R}^{n}$, by the Minkowski inequality

3.1 $$\begin{aligned} \begin{aligned}[b] \Vert H_{\Phi,A}f \Vert _{L^{q_{2}}(B;w)} &= \biggl( \int_{B} \biggl\vert \int_{\mathbb {R}^{n}}\frac{\Phi(y)}{\vert y\vert^{n}}f\bigl(A(y) x\bigr)\,dy \biggr\vert ^{q_{2}}w(x)\,dx \biggr)^{1/q_{2}} \\ &\le \int_{\mathbb {R}^{n}}\frac{ \vert \Phi(y) \vert }{ \vert y \vert ^{n}} \biggl( \int_{B} \bigl\vert f\bigl(A(y) x\bigr) \bigr\vert ^{q_{2}}w(x)\,dx \biggr)^{1/q_{2}}\,dy. \end{aligned} \end{aligned}$$

In view of the condition $q_{1}> q_{2}r_{w}/(r_{w}-1)$, there exists $1< r< r_{w}$ such that $q_{1}=q_{2}r^{\prime}=q_{2}r/(r-1)$. An application of the Hölder inequality and the reverse Hölder condition yields

$$\begin{aligned} \begin{aligned} \bigl\Vert f\bigl(A(y)\cdot\bigr) \bigr\Vert _{L^{q_{2}}(B;w)}&\le \biggl( \int _{B} \bigl\vert f\bigl(A(y) x\bigr) \bigr\vert ^{q_{1}}\,dx \biggr)^{1/q_{1}} \biggl( \int_{B}w(x)^{r}\,dx \biggr)^{1/(rq_{2})} \\ &\preceq \bigl\vert \det A^{-1}(y) \bigr\vert ^{1/q_{1}} \vert B \vert ^{-1/q_{1}}w(B)^{1/q_{2}} \biggl( \int_{A(y)B} \bigl\vert f(x) \bigr\vert ^{q_{1}}\,dx \biggr)^{1/q_{1}}. \end{aligned} \end{aligned}$$

By virtue of Proposition 2.4, one has

3.2 $$\begin{aligned} \begin{aligned}[b] & \biggl( \int_{A(y)B} \bigl\vert f(x) \bigr\vert ^{q_{1}}\,dx \biggr)^{1/q_{1}} \\ &\quad \preceq \bigl\vert B\bigl(0, \bigl\Vert A(y) \bigr\Vert R\bigr) \bigr\vert ^{1/q_{1}} \biggl(\frac{1}{w(B(0, \Vert A(y) \Vert R))} \int _{B(0, \Vert A(y) \Vert R)} \bigl\vert f(x) \bigr\vert ^{q_{1}}w(x)\,dx \biggr)^{1/q_{1}} \\ &\quad \preceq \bigl\Vert A(y) \bigr\Vert ^{n/q_{1}} \bigl\vert B(0,R) \bigr\vert ^{1/q_{1}}w\bigl(B\bigl(0, \bigl\Vert A(y) \bigr\Vert R \bigr)\bigr)^{\lambda} \Vert f \Vert _{\dot{M}^{q_{1},\lambda}(\mathbb {R}^{n},w)}, \end{aligned} \end{aligned}$$

which suggests that

3.3 $$\begin{aligned} \begin{aligned}[b] & \bigl\Vert f\bigl(A(y)\cdot \bigr) \bigr\Vert _{L^{q_{2}}(B;w)} \\ &\quad \preceq \bigl\vert \det A^{-1}(y) \bigr\vert ^{1/q_{1}} \bigl\Vert A(y) \bigr\Vert ^{n/q_{1}}w(B)^{1/q_{2}}w\bigl(B\bigl(0, \bigl\Vert A(y) \bigr\Vert R\bigr)\bigr)^{\lambda} \Vert f \Vert _{\dot{M}^{q_{1},\lambda}(\mathbb {R}^{n},w)}. \end{aligned} \end{aligned}$$

We thus conclude from (3.1) and (3.3) that

3.4 $$ \begin{aligned}[b] & \Vert H_{\Phi,A}f \Vert _{\dot{M}^{q_{2},\lambda}(\mathbb {R}^{n};w)} \\ &\quad \preceq \Vert f \Vert _{\dot{M}^{q_{1},\lambda }(\mathbb {R}^{n},w)} \int_{\mathbb {R}^{n}}\frac{ \vert \Phi(y) \vert }{ \vert y \vert ^{n}} \bigl\vert \det A^{-1}(y) \bigr\vert ^{1/q_{1}} \bigl\Vert A(y) \bigr\Vert ^{n/q_{1}} \biggl(\frac{w(B(0, \Vert A(y) \Vert R))}{w(B(0,R))} \biggr)^{\lambda}\,dy \\ &\quad \preceq \Vert f \Vert _{\dot{M}^{q_{1},\lambda}(\mathbb {R}^{n},w)} \\ &\qquad{} \times \biggl( \int_{ \Vert A(y) \Vert < 1}\frac{ \vert \Phi(y) \vert }{ \vert y \vert ^{n}} \bigl\vert \det A^{-1}(y) \bigr\vert ^{1/q_{1}} \bigl\Vert A(y) \bigr\Vert ^{n/q_{1}} \biggl(\frac{w(B(0, \Vert A(y) \Vert R))}{w(B(0,R))} \biggr)^{\lambda}\,dy \\ &\qquad{} + \int_{ \Vert A(y) \Vert \ge1}\frac{ \vert \Phi (y) \vert }{ \vert y \vert ^{n}} \bigl\vert \det A^{-1}(y) \bigr\vert ^{1/q_{1}} \bigl\Vert A(y) \bigr\Vert ^{n/q_{1}} \biggl(\frac{w(B(0, \Vert A(y) \Vert R))}{w(B(0,R))} \biggr)^{\lambda}\,dy \biggr). \end{aligned} $$

Since $\lambda<0$, Proposition 2.3 implies that, if $\Vert A(y) \Vert <1$,

3.5 $$\begin{aligned} \biggl(\frac{w(B(0, \Vert A(y) \Vert R))}{w(B(0,R))} \biggr)^{\lambda}\preceq \biggl( \frac{ \vert B(0, \Vert A(y) \Vert R) \vert }{ \vert B(0,R) \vert } \biggr)^{\lambda}= \bigl\Vert A(y) \bigr\Vert ^{n\lambda}, \end{aligned}$$

and, if $\Vert A(y) \Vert \ge1$,

3.6 $$\begin{aligned} \biggl(\frac{w(B(0, \Vert A(y) \Vert R))}{w(B(0,R))} \biggr)^{\lambda} \preceq \biggl( \frac{ \vert B(0, \Vert A(y) \Vert R) \vert }{ \vert B(0,R) \vert } \biggr)^{\lambda (\delta-1)/\delta}= \bigl\Vert A(y) \bigr\Vert ^{n\lambda(\delta -1)/\delta}, \end{aligned}$$

for any $1<\delta<r_{w}$.

Therefore, from (3.4)-(3.6) it is easy to see that, for any $1<\delta<r_{w}$,

$$ \begin{aligned}[b] & \Vert H_{\Phi,A}f \Vert _{\dot{M}^{q_{2},\lambda}(\mathbb {R}^{n};w)} \\ &\quad \preceq \Vert f \Vert _{\dot{M}^{q_{1},\lambda}(\mathbb {R}^{n},w)} \biggl( \int_{ \Vert A(y) \Vert < 1}\frac{ \vert \Phi(y) \vert }{ \vert y \vert ^{n}} \bigl\vert \det A^{-1}(y) \bigr\vert ^{1/q_{1}} \bigl\Vert A(y) \bigr\Vert ^{n\lambda +n/q_{1}}\,dy \\ &\qquad{} + \int_{ \Vert A(y) \Vert \ge1}\frac{ \vert \Phi (y) \vert }{ \vert y \vert ^{n}} \bigl\vert \det A^{-1}(y) \bigr\vert ^{1/q_{1}} \bigl\Vert A(y) \bigr\Vert ^{n/q_{1}+n\lambda(\delta-1)/\delta}\,dy \biggr). \end{aligned} $$

The proof of Theorem 3.1 is completed.

### Proof of Theorem 3.2

In view of the Minkowski inequality, change of variables and Proposition 2.7, we have

$$\begin{aligned} \begin{aligned} & \biggl(\frac{1}{ v{(B(0,R))^{1+\lambda q}}} \int_{B(0,R)} \vert H_{\Phi, A}f \vert ^{q} v(x)\,dx \biggr)^{1/q} \\ &\quad \preceq v\bigl(B(0,R)\bigr)^{-(\lambda+1/q)} \int_{\mathbb {R}^{n}}\frac{ \vert \Phi(y) \vert }{ \vert y \vert ^{n}} \biggl( \int _{B} \bigl\vert f\bigl(A(y) x\bigr) \bigr\vert ^{q}v(x)\,dx \biggr)^{1/q}\,dy \\ &\quad \simeq v\bigl(B(0,R)\bigr)^{-(\lambda+1/q)} \\ &\qquad{} \times \int_{\mathbb {R}^{n}}\frac{ \vert \Phi(y) \vert }{ \vert y \vert ^{n}} \bigl\vert \det A^{-1}(y) \bigr\vert ^{1/q} \biggl( \int_{A(y)B} \bigl\vert f(x) \bigr\vert ^{q}v \bigl(A^{-1}(y)x\bigr)\,dx \biggr)^{1/q}\,dy \\ &\quad \preceq \Vert f \Vert _{\dot{M}^{q,\lambda}(\mathbb {R}^{n},v)} \\ &\qquad{} \times \textstyle\begin{cases} \int_{\mathbb {R}^{n}}\frac{ \vert \Phi(y) \vert }{ \vert y \vert ^{n}} \vert \det A^{-1}(y) \vert ^{1/q} \Vert A(y) \Vert ^{(n+\alpha)(\lambda+1/q)} \Vert A^{-1}(y) \Vert ^{\alpha/q}\,dy & \text{if } \alpha>0,\\ \int_{\mathbb {R}^{n}}\frac{ \vert \Phi(y) \vert }{ \vert y \vert ^{n}} \vert \det A^{-1}(y) \vert ^{1/q} \Vert A(y) \Vert ^{n(\lambda+1/q)+\alpha\lambda}\,dy & \text{if } \alpha\le0. \end{cases}\displaystyle \end{aligned} \end{aligned}$$

Therefore, we conclude that

$$\begin{aligned} \begin{aligned} \Vert H_{\Phi, A} \Vert _{\dot{M}^{q,\lambda}(\mathbb {R}^{n},v)\rightarrow\dot{M}^{q,\lambda}(\mathbb {R}^{n},v)}& \preceq \textstyle\begin{cases} K_{2} & \text{if } \alpha>0,\\ K_{3} & \text{if } \alpha\le0. \end{cases}\displaystyle \end{aligned} \end{aligned}$$

Thus we finish the proof of Theorem 3.2.

### Proof of Theorem 3.3

If $\Vert A^{-1}(y) \Vert \preceq \Vert A(y) \Vert ^{-1}$, we infer from (2.2) that

3.7 $$\begin{aligned} \begin{aligned} \bigl\Vert A(y) \bigr\Vert ^{-n}\simeq \bigl\vert \det A^{-1}(y) \bigr\vert \simeq \bigl\Vert A^{-1}(y) \bigr\Vert ^{n}. \end{aligned} \end{aligned}$$

Here we will prove the necessary part of Theorem 3.3, as the sufficient part can easily be obtained from Theorem 3.2. We divide our proof into the following two cases.

Case 1. If $-1/q<\lambda<\infty$.

In this case, we select $f_{0}\in \dot{M}^{p,\lambda}(\mathbb {R}^{n};v)$ such that $f_{0}(x)= \vert x \vert ^{(n+\alpha)\lambda}$. It is easy to see that $\Vert f_{0} \Vert _{\dot{M}^{p,\lambda}(\mathbb {R}^{n};v)}= \vert S^{n-1} \vert ^{-\lambda}(n+\alpha )^{\lambda}(1+\lambda q)^{-1/q}$, where $\vert S^{n-1} \vert $ denotes the volume of unit sphere $S^{n-1}$.

On the other hand, making use of the fact that $(n+\alpha)\lambda<0$, we obtain

$$\begin{aligned} \begin{aligned} H_{\Phi, A}f_{0}(x)&= \int_{\mathbb {R}^{n}}\frac{\Phi(y)}{ \vert y \vert ^{n}} \bigl\vert A(y) x \bigr\vert ^{(n+\alpha)\lambda }\,dy \\ &\succeq \vert x \vert ^{(n+\alpha)\lambda} \int_{\mathbb {R}^{n}}\frac{\Phi(y)}{ \vert y \vert ^{n}} \bigl\Vert A(y) \bigr\Vert ^{(n+\alpha)\lambda}\,dy, \end{aligned} \end{aligned}$$

this implies that

$$\begin{aligned} \Vert H_{\Phi, A} \Vert _{\dot {M}^{q,\lambda}(\mathbb {R}^{n},v)\rightarrow\dot{M}^{q,\lambda }(\mathbb {R}^{n},v)}\succeq \int_{\mathbb {R}^{n}}\frac{\Phi(y)}{ \vert y \vert ^{n}} \bigl\Vert A(y) \bigr\Vert ^{(n+\alpha )\lambda}\,dy, \end{aligned}$$

as required.

Case 2. If $\lambda=-1/q$, then, for $0<\epsilon<1$, we take

$$\begin{aligned} f_{\epsilon}(x)= \vert x \vert ^{-(n+\alpha )/q-\epsilon}\chi_{\{ \vert x \vert >1\}}. \end{aligned}$$

A simple computation yields $\Vert f_{\epsilon} \Vert _{L^{q}(\mathbb {R}^{n};v)}^{q}=\frac{ \vert S^{n-1} \vert }{\epsilon q}$. Now, by definition

$$\begin{aligned} \begin{aligned}H_{\Phi,A}(f_{\epsilon}) (x)&= \int_{\mathbb {R}^{n}}\frac {\Phi(y)}{ \vert y \vert ^{n}} \bigl\vert A(y) x \bigr\vert ^{-(n+\alpha)/q-\epsilon}\chi_{\{ \vert A(y) x \vert >1\} }\,dy \\ &\succeq \biggl( \int_{ \Vert A(y) \Vert \succeq1/ \vert x \vert }\frac{\Phi(y)}{ \vert y \vert ^{n}} \bigl\Vert A(y) \bigr\Vert ^{-(n+\alpha)/q-\epsilon} \biggr) \vert x \vert ^{-(n+\alpha)/q-\epsilon}. \end{aligned} \end{aligned}$$

Now,

$$\begin{aligned} \begin{aligned}& \bigl\Vert H_{\Phi,A}(f_{\epsilon}) \bigr\Vert _{L^{q}(\mathbb {R}^{n},v)}^{q} \\ &\quad \succeq \int_{ \vert x \vert >1} \biggl( \vert x \vert ^{-(n+\alpha)/q-\epsilon} \int _{ \Vert A(y) \Vert \succeq1/ \vert x \vert }\frac{\Phi(y)}{ \vert y \vert ^{n}} \bigl\Vert A(y) \bigr\Vert ^{-(n+\alpha)/q-\epsilon} \biggr)^{q}v(x)\,dx \\ &\quad \succeq \int_{ \vert x \vert >\frac{1}{\epsilon}} \vert x \vert ^{-n-\epsilon q}\,dx \biggl( \int_{ \Vert A(y) \Vert \succeq\epsilon}\frac{\Phi(y)}{ \vert y \vert ^{n}} \bigl\Vert A(y) \bigr\Vert ^{-(n+\alpha)/q-\epsilon }\,dy \biggr)^{q} \\ &\quad = \biggl( \int_{ \Vert A(y) \Vert \succeq\epsilon}\frac {\Phi(y)}{ \vert y \vert ^{n}} \bigl\Vert A(y) \bigr\Vert ^{-(n+\alpha)/q-\epsilon}\,dy \biggr)^{q}\bigl(\epsilon^{\epsilon}\bigr)^{q} \Vert f_{\epsilon} \Vert _{L^{q}(\mathbb {R}^{n},v)}^{q}, \end{aligned} \end{aligned}$$

by letting $\epsilon\rightarrow0$, we have

$$\begin{aligned} \Vert H_{\Phi} \Vert _{{L^{q}(\mathbb {R}^{n},v)}\rightarrow{L^{q}(\mathbb {R}^{n},v)}}\succeq \int_{\mathbb {R}^{n}}\frac{\Phi(y)}{ \vert y \vert ^{n}} \bigl\Vert A(y) \bigr\Vert ^{-(n+\alpha)/q}\,dy. \end{aligned}$$

With this we complete the proof of Theorem 3.3.

## Bounds for $H_{\Phi,A}^{b}$ on weighted central Morrey space

### Main results

We now present the main results for this section.

#### Theorem 4.1

*Let* $1\le q<\infty$, $1\le s< q_{1}<\infty$, $1/s=1/q_{1}+1/q_{2}$, *and* $\lambda<0$. *Suppose that* $w\in A_{1}$ *with the critical index* $r_{w}$ *for the reverse Hölder condition and suppose that* $s>qr_{w}/(r_{w}-1)$.

*Then for any* $1<\delta<r_{w}$

$$\begin{aligned} \begin{aligned} \bigl\Vert H_{\Phi,A}^{b}f \bigr\Vert _{\dot{M}^{q,\lambda}(\mathbb {R}^{n};w)}&\preceq K_{5} \Vert b \Vert _{C\dot{M}O^{q_{2}}(\mathbb {R}^{n},w)} \Vert f \Vert _{\dot{M}^{q_{1},\lambda}(\mathbb {R}^{n},w)}, \end{aligned} \end{aligned}$$

*where*

$$\begin{aligned} \begin{aligned} K_{5}&= \int_{ \Vert A(y) \Vert < 1}\frac{ \vert \Phi (y) \vert \vert \det A^{-1}(y) \vert ^{1/q_{1}}}{ \vert y \vert ^{n} \Vert A(y) \Vert ^{-n\lambda-n/q_{1}}} \biggl(1+\frac{ \vert \det A^{-1}(y) \vert ^{1/q_{2}}}{ \Vert A(y) \Vert ^{-n/q_{2}}} \biggr)\log\frac{2}{ \Vert A(y) \Vert }\,dy \\ &\quad{} + \int_{ \Vert A(y) \Vert \ge1}\frac{ \vert \Phi (y) \vert \vert \det A^{-1}(y) \vert ^{1/q_{1}}}{ \vert y \vert ^{n} \Vert A(y) \Vert ^{-n/q_{1}-n\lambda (\delta-1)/\delta}} \biggl(1+\frac{ \vert \det A^{-1}(y) \vert ^{1/q_{2}}}{ \Vert A(y) \Vert ^{-n/q_{2}}} \biggr)\log2 \bigl\Vert A(y) \bigr\Vert \,dy. \end{aligned} \end{aligned}$$

Instead of general weights, when dealing with power weights, we have the following results.

#### Theorem 4.2

*Let* $1\le q< q_{1}<\infty$, $1/q=1/q_{1}+1/q_{2}$, $-1/q\le\lambda<0$. *Then*:

(i) *If* $0<\alpha<\infty$,
  $$\begin{aligned} \begin{aligned} \bigl\Vert H_{\Phi,A}^{b}f \bigr\Vert _{\dot{M}^{q,\lambda}(\mathbb {R}^{n}; v)}&\preceq K_{6} \Vert b \Vert _{C\dot {M}O^{q_{2}}(\mathbb {R}^{n}, v)} \Vert f \Vert _{\dot{M}^{q_{1},\lambda}(\mathbb {R}^{n}, v)}, \end{aligned} \end{aligned}$$
  *where*
  $$ \begin{aligned}[b] K_{6}&= \int_{\mathbb {R}^{n}}\frac{ \vert \Phi(y) \vert \vert \det A^{-1}(y) \vert ^{\frac{1}{q_{1}}} \Vert A^{-1}(y) \Vert ^{\frac{\alpha}{q_{1}}}}{ \vert y \vert ^{n} \Vert A(y) \Vert ^{-(n+\alpha) (\lambda+\frac{1}{q_{1}})}} \biggl(1+\frac{ \vert \det A^{-1}(y) \vert ^{\frac{1}{q_{2}}} \Vert A^{-1}(y) \Vert ^{\frac{\alpha}{q_{2}}}}{ \Vert A(y) \Vert ^{-\frac {n+\alpha}{q_{2}}}} \biggr) \\ &\quad {}\times\biggl(\log\frac{2}{ \Vert A(y) \Vert }\chi_{\{ \Vert A(y) \Vert < 1\}}+\log2 \bigl\Vert A(y) \bigr\Vert \chi _{\{ \Vert A(y) \Vert \ge1\}} \biggr)\,dy . \end{aligned} $$
(ii) *If* $-n<\alpha\le0$, *then*
  $$\begin{aligned} \begin{aligned} \bigl\Vert H_{\Phi,A}^{b}f \bigr\Vert _{\dot{M}^{q,\lambda}(\mathbb {R}^{n}; v)}&\preceq K_{7} \Vert b \Vert _{C\dot {M}O^{q_{2}}(\mathbb {R}^{n}, v)} \Vert f \Vert _{\dot{M}^{q_{1},\lambda}(\mathbb {R}^{n}, v)}, \end{aligned} \end{aligned}$$
  *where*
  $$ \begin{aligned}[b] K_{7}&= \int_{\mathbb {R}^{n}}\frac{ \vert \Phi(y) \vert \vert \det A^{-1}(y) \vert ^{\frac{1}{q_{1}}}}{ \vert y \vert ^{n} \Vert A(y) \Vert ^{-n(\lambda+\frac {1}{q_{1}})-\alpha\lambda}} \biggl(1+\frac{ \vert \det A^{-1}(y) \vert ^{\frac {1}{q_{2}}}}{ \Vert A(y) \Vert ^{-\frac{n}{q_{2}}}} \biggr) \\ &\quad {}\times \biggl(\log\frac{2}{ \Vert A(y) \Vert }\chi_{\{ \Vert A(y) \Vert < 1\}}+\log2 \bigl\Vert A(y) \bigr\Vert \chi _{\{ \Vert A(y) \Vert \ge1\}} \biggr)\,dy . \end{aligned} $$

More specifically, if $\Vert A^{-1}(y) \Vert $ and $\Vert A(y) \Vert ^{-1}$ are comparable, we obtain the sharp results by decomposing $H_{\phi,A}^{b}$ as follows:

$$\begin{gathered} H_{\Phi,A}^{b,1}f= \int_{ \Vert A(y) \Vert < 1}\frac{\Phi (y)}{ \vert y \vert ^{n}}\bigl(b(x)-b\bigl(A(y) x\bigr) \bigr)f\bigl(A(y) x\bigr)\,dy, \\ H_{\Phi,A}^{b,2}f= \int_{ \Vert A(y) \Vert \ge1}\frac {\Phi(y)}{ \vert y \vert ^{n}}\bigl(b(x)-b\bigl(A(y) x\bigr) \bigr)f\bigl(A(y) x\bigr)\,dy. \end{gathered} $$

#### Theorem 4.3

*Let* $1< q< q_{1}<\infty$, $1/q=1/q_{1}+1/q_{2}$, $-1/q<\lambda<0$, *and* Φ *be a nonnegative function*. *Suppose that there is a positive constant* *C* *independent of* *y* *such that* $\Vert A^{-1}(y) \Vert \le C \Vert A(y) \Vert ^{-1}$ *for all* $y\in\operatorname{supp}(\Phi)$. *In addition*, *if* $\Phi(y)/ \vert y \vert ^{n}$ *is integrable then*:

(i) $H_{\Phi,A}^{b,1}$ *is bounded from* $\dot{M}^{q_{1},\lambda }(\mathbb {R}^{n};v)$ *to* $\dot{M}^{q,\lambda}(\mathbb {R}^{n};v)$ *if and only if*
  $$\begin{aligned} \begin{aligned} K_{8}= \int_{ \Vert A(y) \Vert < 1}\frac{\Phi(y)}{ \vert y \vert ^{n}}\vert \bigl\Vert A(y) \bigr\Vert ^{(n+\alpha)\lambda}\log\frac{2}{ \Vert A(y) \Vert }\,dy< \infty. \end{aligned} \end{aligned}$$
(ii) $H_{\Phi,A}^{b,2}$ *is bounded from* $\dot{M}^{q_{1},\lambda }(\mathbb {R}^{n};v)$ *to* $\dot{M}^{q,\lambda}(\mathbb {R}^{n};v)$ *if and only if*
  $$\begin{aligned} \begin{aligned} K_{9}= \int_{ \Vert A(y) \Vert \ge1}\frac{\Phi(y)}{ \vert y \vert ^{n}}\vert \bigl\Vert A(y) \bigr\Vert ^{(n+\alpha)\lambda}\log2 \bigl\Vert A(y) \bigr\Vert \,dy< \infty. \end{aligned} \end{aligned}$$

#### Remark 4.4

Note that, from the preceding theorem, one cannot deduce $L^{p}(\mathbb {R}^{n};v)$ boundedness for the commutator operator by taking $\lambda=-1/p$, just in the case of Theorem 3.3. This raises an open question regarding the $L^{p}$ boundedness of $H_{\Phi,A}^{b}$, which will be answered later.

#### Remark 4.5

Validity of assumptions in Theorem 4.3 can be justified by Remark 3.4 in third section.

### Proof of Theorem 4.1

As before we fix a ball $B\subset\mathbb {R}^{n}$. Using Minkowski inequality, we obtain

$$\begin{aligned} \begin{aligned}[b] & \bigl\Vert H_{\Phi,A}^{b}f \bigr\Vert _{L^{q}(B;w)} \\ &\quad = \biggl( \int_{B} \biggl\vert \int_{\mathbb {R}^{n}}\frac{\Phi(y)}{\vert y\vert^{n}}\bigl(b(x)-b\bigl(A(y) x\bigr) \bigr)f\bigl(A(y) x\bigr)\,dy \biggr\vert ^{q}w(x)\,dx \biggr)^{1/q} \\ &\quad \le \int_{\mathbb {R}^{n}}\frac{ \vert \Phi(y) \vert }{ \vert y \vert ^{n}} \biggl( \int_{B} \bigl\vert \bigl(b(x)-b\bigl(A(y) x\bigr)\bigr)f \bigl(A(y) x\bigr) \bigr\vert ^{q}w(x)\,dx \biggr)^{1/q}\,dy \\ &\quad \le \int_{\mathbb {R}^{n}}\frac{ \vert \Phi(y) \vert }{ \vert y \vert ^{n}} \biggl( \int_{B} \bigl\vert \bigl(b(x)-b_{B,w}\bigr)f \bigl(A(y) x\bigr) \bigr\vert ^{q}w(x)\,dx \biggr)^{1/q}\,dy \\ &\qquad{} + \int_{\mathbb {R}^{n}}\frac{ \vert \Phi(y) \vert }{ \vert y \vert ^{n}} \biggl( \int_{B} \bigl\vert (b_{B,w}-b_{A(y)B,w})f \bigl(A(y) x\bigr) \bigr\vert ^{q}w(x)\,dx \biggr)^{1/q}\,dy \\ &\qquad{} + \int_{\mathbb {R}^{n}}\frac{ \vert \Phi(y) \vert }{ \vert y \vert ^{n}} \biggl( \int_{B} \bigl\vert \bigl(b\bigl(A(y) x \bigr)-b_{A(y)B,w}\bigr)f\bigl(A(y) x\bigr) \bigr\vert ^{q}w(x)\,dx \biggr)^{1/q}\,dy \\ &\quad =I_{1}+I_{2}+I_{3}. \end{aligned} \end{aligned}$$

Let us start estimating $I_{1}$. For this purpose, we first compute the inner norm $\Vert (b(\cdot)-b_{B,w})f(A(y)\cdot) \Vert _{L^{q}(B;w)}$. The condition $s> qr_{w}/(r_{w}-1)$ implies that there is $1< r< r_{w}$ such that $s=qr^{\prime}=qr/(r-1)$. By the Hölder inequality and the reverse Hölder condition, we obtain

$$\begin{aligned} \begin{aligned} & \bigl\Vert \bigl(b(\cdot)-b_{B,w} \bigr)f\bigl(A(y)\cdot\bigr) \bigr\Vert _{L^{q}(B;w)} \\ &\quad \le \biggl( \int_{B} \bigl\vert \bigl(b(x)-b_{B,w}\bigr)f \bigl(A(y) x\bigr) \bigr\vert ^{s}\,dx \biggr)^{1/s} \biggl( \int_{B}w(x)^{r}\,dx \biggr)^{1/(rq)} \\ &\quad \preceq \vert B \vert ^{-1/s}w(B)^{1/q} \biggl( \int _{B} \bigl\vert \bigl(b(x)-b_{B,w}\bigr)f \bigl(A(y) x\bigr) \bigr\vert ^{s}\,dx \biggr)^{1/s}. \end{aligned} \end{aligned}$$

In view of the condition $1/s=1/q_{1}+1/q_{2}$, we have

4.1 $$\begin{aligned} \begin{aligned}[b] & \bigl\Vert \bigl(b( \cdot)-b_{B,w}\bigr)f\bigl(A(y)\cdot\bigr) \bigr\Vert _{L^{q}(B;w)} \\ &\quad \preceq \vert B \vert ^{-1/s}w(B)^{1/q} \biggl( \int _{B} \bigl\vert b(x)-b_{B,w} \bigr\vert ^{q_{2}}\,dx \biggr)^{1/q_{2}} \biggl( \int_{B} \bigl\vert f\bigl(A(y) x\bigr) \bigr\vert ^{q_{1}}\,dx \biggr)^{1/q_{1}} \\ &\quad \preceq \bigl\vert \det A^{-1}(y) \bigr\vert ^{1/q_{1}} \vert B \vert ^{-1/s}w(B)^{1/q} \\ &\qquad{} \times \biggl( \int_{B} \bigl\vert b(x)-b_{B,w} \bigr\vert ^{q_{2}}\,dx \biggr)^{1/q_{2}} \biggl( \int_{A(y)B} \bigl\vert f(x) \bigr\vert ^{q_{1}}\,dx \biggr)^{1/q_{1}}. \end{aligned} \end{aligned}$$

By virtue of Proposition 2.4, it is easy to see that

4.2 $$\begin{aligned} \begin{aligned} \biggl( \int_{B} \bigl\vert b(x)-b_{B,w} \bigr\vert ^{q_{2}}\,dx \biggr)^{1/q_{2}} &\preceq \vert B \vert ^{1/q_{2}} \Vert b \Vert _{C\dot{M}O^{q_{2}}(\mathbb {R}^{n},w)}. \end{aligned} \end{aligned}$$

Substituting the result from inequalities (3.2) and (4.2) into (4.1), one has

$$\begin{aligned} \begin{aligned} & \bigl\Vert \bigl(b(\cdot)-b_{B,w} \bigr)f\bigl(A(y)\cdot\bigr) \bigr\Vert _{L^{q}(B;w)} \\ &\quad \preceq w(B)^{1/q} \bigl\vert \det A^{-1}(y) \bigr\vert ^{1/q_{2}} \bigl\Vert A(y) \bigr\Vert ^{n/q_{1}}w\bigl(A(y)B \bigr)^{\lambda} \Vert b \Vert _{C\dot{M}O^{q_{2}}(\mathbb {R}^{n},w)} \Vert f \Vert _{\dot{M}^{q_{1},\lambda}(\mathbb {R}^{n},w)}. \end{aligned} \end{aligned}$$

Therefore, we obtain

4.3 $$\begin{aligned} \begin{aligned}[b] I_{1}&\preceq w(B)^{\lambda+1/q} \Vert b \Vert _{C\dot {M}O^{q_{2}}(\mathbb {R}^{n},w)} \Vert f \Vert _{\dot {M}^{q_{1},\lambda}(\mathbb {R}^{n},w)} \\ &\quad{} \times \int_{\mathbb {R}^{n}}\frac{ \vert \Phi(y) \vert }{ \vert y \vert ^{n}} \bigl\vert \det A^{-1}(y) \bigr\vert ^{1/q_{1}} \bigl\Vert A(y) \bigr\Vert ^{n/q_{1}} \biggl(\frac{w(B(0, \Vert A(y) \Vert R))}{w(B(0,R))} \biggr)^{\lambda}\,dy. \end{aligned} \end{aligned}$$

Making use of the inequalities (3.5) and (3.6) into (4.3), we get

$$ \begin{aligned}[b] I_{1}&\preceq w(B)^{\lambda+1/q} \Vert b \Vert _{C\dot {M}O^{q_{2}}(\mathbb {R}^{n},w)} \Vert f \Vert _{\dot {M}^{q_{1},\lambda}(\mathbb {R}^{n},w)} \\ &\quad \times{} \biggl( \int_{ \Vert A(y) \Vert < 1}\frac{ \vert \Phi(y) \vert }{ \vert y \vert ^{n}} \bigl\vert \det A^{-1}(y) \bigr\vert ^{1/q_{1}} \bigl\Vert A(y) \bigr\Vert ^{n\lambda +n/q_{1}}\,dy \\ &\quad{} + \int_{ \Vert A(y) \Vert \ge1}\frac{ \vert \Phi (y) \vert }{ \vert y \vert ^{n}} \bigl\vert \det A^{-1}(y) \bigr\vert ^{1/q_{1}} \bigl\Vert A(y) \bigr\Vert ^{n\lambda (\delta-1)/\delta+n/q_{1}}\,dy \biggr). \end{aligned} $$

Now, we turn to an estimate of $I_{2}$, which can be written as

4.4 $$\begin{aligned} \begin{aligned} I_{2}&= \int_{\mathbb {R}^{n}}\frac{ \vert \Phi(y) \vert }{ \vert y \vert ^{n}} \bigl\Vert f\bigl(A(y)\cdot \bigr) \bigr\Vert _{L^{q}(B;w)} \vert b_{B,w}-b_{A(y)B,w} \vert \,dy. \end{aligned} \end{aligned}$$

Here, the indices *q* and *s* bear the same relationship as observed between $q_{1}$ and $q_{2}$ of Theorem 3.1. Therefore, we infer from (3.3) that

$$\begin{aligned} \begin{aligned}[b] & \bigl\Vert f\bigl(A(y)\cdot\bigr) \bigr\Vert _{L^{q}(B;w)} \\ &\quad \preceq \bigl\vert \det A^{-1}(y) \bigr\vert ^{1/s} \bigl\Vert A(y) \bigr\Vert ^{n/s}w\bigl(B(0,R)\bigr)^{1/q} w \bigl(B\bigl(0, \bigl\Vert A(y) \bigr\Vert R\bigr)\bigr)^{\lambda} \Vert f \Vert _{\dot{M}^{s,\lambda}(\mathbb {R}^{n},w)}. \end{aligned} \end{aligned}$$

Applying the Hölder inequality to $s/q_{1}$ and $s/q_{2}$, we have

4.5 $$\begin{aligned} \begin{aligned}[b] & \bigl\Vert f\bigl(A(y)\cdot \bigr) \bigr\Vert _{L^{q}(B;w)} \\ &\quad \preceq \bigl\vert \det A^{-1}(y) \bigr\vert ^{1/s} \bigl\Vert A(y) \bigr\Vert ^{n/s} w(B)^{1/q}w\bigl(B\bigl(0, \bigl\Vert A(y) \bigr\Vert R\bigr)\bigr)^{\lambda} \Vert f \Vert _{\dot{M}^{q_{1},\lambda}(\mathbb {R}^{n},w)}. \end{aligned} \end{aligned}$$

With the help of (4.5), inequality (4.4) assumes the following form:

$$\begin{aligned} \begin{aligned} I_{2}&\preceq w(B)^{\lambda+1/q} \Vert f \Vert _{\dot {M}^{q_{1},\lambda}(\mathbb {R}^{n},w)} \\ &\quad {}\times \int_{\mathbb {R}^{n}}\frac{ \vert \Phi(y) \vert }{ \vert y \vert ^{n}} \bigl\vert \det A^{-1}(y) \bigr\vert ^{1/s} \bigl\Vert A(y) \bigr\Vert ^{n/s}\frac{w(B(0,\Vert A(y)\Vert R))^{\lambda}}{w(B(0,R))^{\lambda}} \vert b_{B,w}-b_{A(y)B,w} \vert \,dy. \end{aligned} \end{aligned}$$

For $\lambda<0$, the inequalities (3.5) and (3.6) help us to obtain

4.6 $$ \begin{aligned}[b] I_{2}&\preceq w(B)^{\lambda+1/q} \Vert f \Vert _{\dot {M}^{q_{1},\lambda}(\mathbb {R}^{n},w)} \\ &\quad {}\times \biggl( \int_{ \Vert A(y) \Vert < 1}\frac{ \vert \Phi(y) \vert }{ \vert y \vert ^{n}} \bigl\vert \det A^{-1}(y) \bigr\vert ^{1/s} \bigl\Vert A(y) \bigr\Vert ^{n\lambda +n/s} \vert b_{B,w}-b_{A(y)B,w} \vert \,dy \\ &\quad{} + \int_{ \Vert A(y) \Vert \ge1}\frac{ \vert \Phi (y) \vert }{ \vert y \vert ^{n}} \bigl\vert \det A^{-1}(y) \bigr\vert ^{1/s} \bigl\Vert A(y) \bigr\Vert ^{n\lambda (\delta-1)/\delta+n/s} \vert b_{B,w}-b_{A(y)B,w} \vert \,dy \biggr) \\ & =: w(B)^{\lambda+1/q} \Vert f \Vert _{\dot {M}^{q_{1},\lambda}(\mathbb {R}^{n},w)} ( I_{21} +I_{22} ). \end{aligned} $$

For convenience, we denote $\phi(y)=\frac{ \vert \Phi(y) \vert }{ \vert y \vert ^{n}} \vert \det A^{-1}(y) \vert ^{1/s} \Vert A(y) \Vert ^{n(\lambda+1/s)}$. Moreover, for $\Vert A(y) \Vert <1$, there exists $j\in\mathbb {Z}$ such that $2^{-j-1}\le \Vert A(y) \Vert <2^{-j}$. Thus

$$ \begin{aligned} I_{21}&= \int_{ \Vert A(y) \Vert < 1}\phi(y) \Biggl\{ \sum_{i=1}^{j} \vert b_{2^{-i}B,w}-b_{2^{-i+1}B,w} \vert + \vert b_{2^{-j}B,w}-b_{A(y)B,w} \vert \Biggr\} \,dy. \end{aligned} $$

Since $w\in A_{1}$, using the Hölder inequality it is easy to see that

$$ \begin{aligned} \vert b_{2^{-i}B,w}-b_{2^{-i+1}B,w} \vert & \le\frac {1}{w(2^{-i}B)} \int_{2^{-i}B} \bigl\vert b(y)-b_{2^{-i+1}B,w} \bigr\vert w(y)\,dy \\ &\le\frac{w(2^{-i+1}B)}{w(2^{-i}B)} \Vert b \Vert _{C\dot {M}O^{q_{2}}(\mathbb {R}^{n},w)} \\ &\preceq \Vert b \Vert _{C\dot{M}O^{q_{2}}(\mathbb {R}^{n},w)}. \end{aligned} $$

Similarly, $\vert b_{2^{-j}B,w}-b_{A(y)B,w} \vert \preceq \Vert b \Vert _{C\dot{M}O^{q_{2}}(\mathbb {R}^{n},w)}$ and thus

$$ \begin{aligned} I_{21}&\preceq \Vert b \Vert _{C\dot{M}O^{q_{2}}(\mathbb {R}^{n},w)}\sum_{j=0}^{\infty}\int_{2^{-j-1}\le \Vert A(y) \Vert < 2^{-j}}\phi(y) \{j+1 \}\,dy \\ &\preceq \Vert b \Vert _{C\dot{M}O^{q_{2}}(\mathbb {R}^{n},w)}\sum_{j=0}^{\infty}\int_{2^{-j-1}\le \Vert A(y) \Vert < 2^{-j}}\phi(y) \bigl\{ \log2^{j}+1 \bigr\} \,dy \\ &\preceq \Vert b \Vert _{C\dot{M}O^{q_{2}}(\mathbb {R}^{n},w)} \int_{ \Vert A(y) \Vert < 1}\frac{ \vert \Phi (y) \vert }{ \vert y \vert ^{n}} \bigl\vert \det A^{-1}(y) \bigr\vert ^{1/s} \bigl\Vert A(y) \bigr\Vert ^{n(\lambda +1/s)}\log\frac{2}{ \Vert A(y \Vert }\,dy. \end{aligned} $$

Following the same procedure as in the approximation of $I_{21}$, we estimate $I_{22}$ as

$$ \begin{aligned} I_{22}&\preceq \Vert b \Vert _{C\dot{M}O^{q_{2}}(\mathbb {R}^{n},w)} \\ &\quad{} \times \int_{ \Vert A(y) \Vert \ge1}\frac{ \vert \Phi(y) \vert }{ \vert y \vert ^{n}} \bigl\vert \det A^{-1}(y) \bigr\vert ^{1/s} \bigl\Vert A(y) \bigr\Vert ^{n(\lambda (\delta-1)/\delta+1/s)}\log2 \bigl\Vert A(y \bigr\Vert \,dy. \end{aligned} $$

Incorporating the estimates of $I_{21}$ and $I_{22}$ into (4.6), we obtain

$$ \begin{aligned}[b] I_{2}&\preceq w(B)^{\lambda+1/q} \Vert b \Vert _{C\dot {M}O^{q_{2}}(\mathbb {R}^{n},w)} \Vert f \Vert _{\dot {M}^{q_{1},\lambda}(\mathbb {R}^{n},w)} \\ &\quad {}\times \biggl( \int_{ \Vert A(y) \Vert < 1}\frac{ \vert \Phi(y) \vert }{ \vert y \vert ^{n}} \bigl\vert \det A^{-1}(y) \bigr\vert ^{1/s} \bigl\Vert A(y) \bigr\Vert ^{n(\lambda +1/s)}\log\frac{2}{ \Vert A(y \Vert )}\,dy \\ &\quad{} + \int_{ \Vert A(y) \Vert \ge1}\frac{ \vert \Phi (y) \vert }{ \vert y \vert ^{n}} \bigl\vert \det A^{-1}(y) \bigr\vert ^{1/s} \bigl\Vert A(y) \bigr\Vert ^{n(\lambda (\delta-1)/\delta+1/s)}\log2 \bigl\Vert A(y) \bigr\Vert \,dy \biggr). \end{aligned} $$

It remains to approximate $I_{3}$. For this purpose, we infer from (4.1) that

4.7 $$\begin{aligned} \begin{aligned}[b] & \bigl\Vert \bigl(b\bigl(A(y) \cdot\bigr)-b_{A(y)B,w}\bigr)f\bigl( A(y)\cdot\bigr) \bigr\Vert _{L^{q}(B;w)} \\ &\quad \preceq \bigl\vert \det A^{-1}(y) \bigr\vert ^{1/q_{1}} \vert B \vert ^{-1/s}w(B)^{1/q} \\ &\qquad{} \times \biggl( \int_{B} \bigl\vert b\bigl(A(y)x\bigr)-b_{A(y)B,w} \bigr\vert ^{q_{2}}\,dx \biggr)^{1/q_{2}} \biggl( \int_{A(y)B} \bigl\vert f(x) \bigr\vert ^{q_{1}}\,dx \biggr)^{1/q_{1}}. \end{aligned} \end{aligned}$$

Making use of Proposition 2.4, one can obtain

4.8 $$\begin{aligned} \begin{aligned}[b] & \biggl( \int_{B} \bigl\vert b\bigl(A(y)x\bigr)-b_{A(y)B,w} \bigr\vert ^{q_{2}}\,dx \biggr)^{1/q_{2}} \\ &\quad = \bigl\vert \det A^{-1}(y) \bigr\vert ^{1/q_{2}} \biggl( \int_{A(y)B} \bigl\vert b(x)-b_{A(y)B,w} \bigr\vert ^{q_{2}}\,dx \biggr)^{1/q_{2}} \\ &\quad = \bigl\vert \det A^{-1}(y) \bigr\vert ^{1/q_{2}} \bigl\Vert A(y) \bigr\Vert ^{n/q_{2}} \bigl\vert B(0,R) \bigr\vert ^{1/q_{2}} \Vert b \Vert _{C\dot{M}O^{q_{2}}(\mathbb {R}^{n},w)}. \end{aligned} \end{aligned}$$

In view of (3.2), (4.7) and (4.8), it is easy to verify that

$$\begin{aligned} \begin{aligned}[b] & \bigl\Vert \bigl(b\bigl(A(y)\cdot \bigr)-b_{A(y)B,w}\bigr)f\bigl( A(y)\cdot\bigr) \bigr\Vert _{L^{q}(B;w)} \\ &\quad \preceq w(B)^{1/q} \bigl\vert \det A^{-1}(y) \bigr\vert ^{1/s} \bigl\Vert A(y) \bigr\Vert ^{n/s}w\bigl(A(y)B \bigr)^{\lambda} \Vert b \Vert _{C\dot{M}O^{q_{2}}(\mathbb {R}^{n},w)} \Vert f \Vert _{\dot{M}^{q_{1},\lambda}(\mathbb {R}^{n},w)}. \end{aligned} \end{aligned}$$

We thus obtain

$$\begin{aligned} \begin{aligned}[b] I_{3}&\preceq w(B)^{\lambda+1/q} \Vert b \Vert _{C\dot {M}O^{q_{2}}(\mathbb {R}^{n},w)} \Vert f \Vert _{\dot {M}^{q_{1},\lambda}(\mathbb {R}^{n},w)} \\ &\quad{} \times \int_{\mathbb {R}^{n}}\frac{ \vert \Phi(y) \vert }{ \vert y \vert ^{n}} \bigl\vert \det A^{-1}(y) \bigr\vert ^{1/s} \bigl\Vert A(y) \bigr\Vert ^{n/s} \biggl(\frac{w(B(0, \Vert A(y) \Vert R))}{w(B(0,R))} \biggr)^{\lambda}\,dy. \end{aligned} \end{aligned}$$

Finally, inequalities (3.5) and (3.6) lead to

$$ \begin{aligned}[b] I_{3}&\preceq w(B)^{\lambda+1/q} \Vert b \Vert _{C\dot {M}O^{q_{2}}(\mathbb {R}^{n},w)} \Vert f \Vert _{\dot {M}^{q_{1},\lambda}(\mathbb {R}^{n},w)} \\ &\quad{}\times \biggl( \int_{ \Vert A(y) \Vert < 1}\frac{ \vert \Phi(y) \vert }{ \vert y \vert ^{n}} \bigl\vert \det A^{-1}(y) \bigr\vert ^{1/s} \bigl\Vert A(y) \bigr\Vert ^{n\lambda +n/s}\,dy \\ &\quad{} + \int_{ \Vert A(y) \Vert \ge1}\frac{ \vert \Phi (y) \vert }{ \vert y \vert ^{n}} \bigl\vert \det A^{-1}(y) \bigr\vert ^{1/s} \bigl\Vert A(y) \bigr\Vert ^{n\lambda (\delta-1)/\delta+n/s}\,dy \biggr), \end{aligned} $$

for any $1<\delta<r_{w}$.

Combining the estimates for $I_{1}$, $I_{2}$, and $I_{3}$, we obtain

$$\begin{aligned} \begin{aligned} \bigl\Vert H_{\Phi,A}^{b}f \bigr\Vert _{\dot {M}^{q,\lambda}(\mathbb {R}^{n};w)}&\preceq K_{5} \Vert b \Vert _{C\dot{M}O^{q_{2}}(\mathbb {R}^{n},w)} \Vert f \Vert _{\dot {M}^{q_{1},\lambda}(\mathbb {R}^{n},w)}. \end{aligned} \end{aligned}$$

This completes the proof of Theorem 4.1.

### Proof of Theorem 4.2

(i) As in the previous theorem

$$\begin{aligned} \bigl\Vert H_{\Phi,A}^{b}f \bigr\Vert _{L^{q}(B; v)}\le J_{1}+J_{2}+J_{3}, \end{aligned}$$

where $J_{1}$, $J_{2}$ and $J_{3}$ assume the form of $I_{1}$, $I_{2}$ and $I_{3}$, respectively, but with $w(\cdot)$ is replaced by $v(\cdot)$.

An application of the Hölder inequality and change of variables yields

$$\begin{aligned} \begin{aligned}[b] J_{1}&\le \int_{\mathbb {R}^{n}}\frac{ \vert \Phi(y) \vert }{ \vert y \vert ^{n}} \biggl( \int_{B} \bigl\vert b(x)-b_{B, v} \bigr\vert ^{q_{2}} v(x)\,dx \biggr)^{1/q_{2}} \biggl( \int_{B} \bigl\vert f\bigl(A(y) x\bigr) \bigr\vert ^{q_{1}} v(x)\,dx \biggr)^{1/q_{1}}\,dy \\ &\le v(B)^{1/q_{2}} \Vert b \Vert _{C\dot{M}O^{q_{2}}(\mathbb {R}^{n}, v)} \\ &\quad{} \times \int_{\mathbb {R}^{n}}\frac{ \vert \Phi(y) \vert }{ \vert y \vert ^{n}} \bigl\vert \det A^{-1}(y) \bigr\vert ^{1/q_{1}} \biggl( \int_{A(y)B} \bigl\vert f( x) \bigr\vert ^{q_{1}} v \bigl(A^{-1}(y) x\bigr)\,dx \biggr)^{1/q_{1}}\,dy. \end{aligned} \end{aligned}$$

In view of Proposition 2.7 it is easy to see that

$$\begin{aligned} \begin{aligned} J_{1}&\preceq v(B)^{\lambda+1/q} \Vert b \Vert _{C\dot {M}O^{q_{2}}(\mathbb {R}^{n}, v)} \Vert f \Vert _{\dot {M}^{q_{1},\lambda}(\mathbb {R}^{n}, v)} \\ &\quad{} \times \int_{\mathbb {R}^{n}}\frac{ \vert \Phi(y) \vert }{ \vert y \vert ^{n}} \bigl\vert \det A^{-1}(y) \bigr\vert ^{1/q_{1}} \bigl\Vert A(y) \bigr\Vert ^{(n+\alpha)(\lambda+1/q_{1})} \bigl\Vert A^{-1}(y) \bigr\Vert ^{\alpha/q_{1}}\,dy. \end{aligned} \end{aligned}$$

The expression for $J_{2}$ can be written as

4.9 $$\begin{aligned} \begin{aligned} J_{2}&= \int_{\mathbb {R}^{n}}\frac{ \vert \Phi(y) \vert }{ \vert y \vert ^{n}} \bigl\Vert f\bigl(A(y)\cdot \bigr) \bigr\Vert _{L^{q}(B;v)} \vert b_{B,v}-b_{A(y)B,v} \vert \,dy. \end{aligned} \end{aligned}$$

In order to estimate $J_{2}$ we first compute $\Vert f(A(y)\cdot ) \Vert _{L^{q}(B; v)}$. For this purpose a change of variables following the Hölder inequality and Proposition 2.7 gives us

$$\begin{aligned} \begin{aligned} & \bigl\Vert f\bigl(A(y)\cdot\bigr) \bigr\Vert _{L^{q}(B; v)} \\ &\quad = \biggl( \int _{B} \bigl\vert f\bigl(A(y) x\bigr) \bigr\vert ^{q} v(x)\,dx \biggr)^{1/q} \\ &\quad = \bigl\vert \det A^{-1}(y) \bigr\vert ^{1/q} \biggl( \int _{A(y)B} \bigl\vert f( x) \bigr\vert ^{q} v \bigl(A^{-1}(y)x\bigr)\,dx \biggr)^{1/q} \\ &\quad \preceq \bigl\vert \det A^{-1}(y) \bigr\vert ^{1/q} \bigl\Vert A^{-1}(y) \bigr\Vert ^{\alpha/q} \Vert f \Vert _{L^{q}(A(y)B; v)} \\ &\quad \preceq \bigl\vert \det A^{-1}(y) \bigr\vert ^{1/q} \bigl\Vert A^{-1}(y) \bigr\Vert ^{\alpha/q} \Vert f \Vert _{L^{q_{1}}(A(y)B; v)}v\bigl(A(y)B\bigr)^{1/q_{2}} \\ &\quad \preceq v(B)^{\lambda+1/q} \bigl\vert \det A^{-1}(y) \bigr\vert ^{1/q} \bigl\Vert A(y) \bigr\Vert ^{(n+\alpha)(\lambda+1/q)} \bigl\Vert A^{-1}(y) \bigr\Vert ^{\alpha/q} \Vert f \Vert _{\dot {M}^{q_{1},\lambda}(\mathbb {R}^{n},v)}. \end{aligned} \end{aligned}$$

Therefore (4.9) becomes

$$\begin{aligned} \begin{aligned} J_{2}&\preceq v(B)^{\lambda+1/q} \Vert f \Vert _{\dot {M}^{q_{1},\lambda}(\mathbb {R}^{n}, v)} \\ &\quad{} \times \int_{\mathbb {R}^{n}}\frac{ \vert \Phi(y) \vert \vert \det A^{-1}(y) \vert ^{1/q} \Vert A^{-1}(y) \Vert ^{\alpha/q}}{ \vert y \vert ^{n} \Vert A(y) \Vert ^{-(n+\alpha)(\lambda+1/q)}} \vert b_{B, v}-b_{A(y)B, v} \vert \,dy. \end{aligned} \end{aligned}$$

By denoting $\psi(y)=\frac{ \vert \Phi(y) \vert \vert \det A^{-1}(y) \vert ^{1/q} \Vert A^{-1}(y) \Vert ^{\alpha/q}}{ \vert y \vert ^{n} \Vert A(y) \Vert ^{-(n+\alpha)(\lambda+1/q)}}$, we decompose $J_{2}$ as

$$ \begin{aligned}[b] J_{2}&\preceq v(B)^{\lambda+1/q} \Vert f \Vert _{\dot {M}^{q_{1},\lambda}(\mathbb {R}^{n}, v)} \\ &\quad {}\times \biggl( \int_{ \Vert A(y) \Vert < 1}\psi(y) \vert b_{B, v}-b_{A(y)B, v} \vert \,dy + \int_{ \Vert A(y) \Vert \ge1}\psi(y) \vert b_{B, v}-b_{A(y)B, v} \vert \,dy \biggr) \\ &=v(B)^{\lambda+1/q} \Vert f \Vert _{\dot{M}^{q_{1},\lambda }(\mathbb {R}^{n}, v)}(J_{21}+J_{22}). \end{aligned} $$

Again, we arrive at the same point as reached in (4.6) with $w(\cdot)$ replaced by $v(\cdot)$. Therefore, performing in a way similar to that point forward we estimate $J_{2}$ as

$$ \begin{aligned}[b] J_{2}&\preceq v(B)^{\lambda+1/q} \Vert b \Vert _{C\dot {M}O^{q_{2}}(\mathbb {R}^{n}, v)} \Vert f \Vert _{\dot {M}^{q_{1},\lambda}(\mathbb {R}^{n}, v)} \\ &\quad {}\times \biggl( \int_{ \Vert A(y) \Vert < 1}\frac{ \vert \Phi(y) \vert \vert \det A^{-1}(y) \vert ^{1/q} \Vert A^{-1}(y) \Vert ^{\alpha/q}}{ \vert y \vert ^{n} \Vert A(y) \Vert ^{-(n+\alpha)(\lambda+1/q)}} \log\frac{2}{ \Vert A(y )\Vert }\,dy \\ &\quad{} + \int_{ \Vert A(y) \Vert \ge1}\frac{ \vert \Phi (y) \vert \vert \det A^{-1}(y) \vert ^{1/q} \Vert A^{-1}(y) \Vert ^{\alpha/q}}{ \vert y \vert ^{n} \Vert A(y) \Vert ^{-(n+\alpha )(\lambda+1/q)}}\log2 \bigl\Vert A(y)\bigr\Vert \,dy \biggr). \end{aligned} $$

It remains to estimate $J_{3}$. For this purpose we proceed as follows:

$$\begin{aligned} & \bigl\Vert \bigl(b\bigl(A(y)\cdot \bigr)-b_{A(y)B,v}\bigr)f\bigl(A(y)\cdot\bigr) \bigr\Vert _{L^{q}(B; v)} \\ &\quad = \biggl( \int_{B} \bigl\vert \bigl(b\bigl(A(y)x\bigr)-b_{A(y)B,v} \bigr)f\bigl(A(y) x\bigr) \bigr\vert ^{q} v(x)\,dx \biggr)^{1/q} \\ &\quad = \bigl\vert \det A^{-1}(y) \bigr\vert ^{1/q} \biggl( \int _{A(y)B} \bigl\vert \bigl(b(x)-b_{A(y)B,v}\bigr)f( x) \bigr\vert ^{q} v\bigl(A^{-1}(y)x\bigr)\,dx \biggr)^{1/q} \\ &\quad \preceq \bigl\vert \det A^{-1}(y) \bigr\vert ^{1/q} \bigl\Vert A^{-1}(y) \bigr\Vert ^{\alpha/q} \bigl\Vert \bigl(b(\cdot )-b_{A(y)B,v}\bigr)f(\cdot) \bigr\Vert _{L^{q}(A(y)B; v)} \\ &\quad \preceq \bigl\vert \det A^{-1}(y) \bigr\vert ^{1/q} \bigl\Vert A^{-1}(y) \bigr\Vert ^{\alpha/q} \Vert b-b_{A(y)B,v} \Vert _{L^{q_{2}}(A(y)B; v)} \Vert f \Vert _{L^{q_{1}}(A(y)B; v)} \\ &\quad \preceq v(B)^{\lambda+1/q} \Vert b \Vert _{C\dot {M}O^{q_{2}}(\mathbb {R}^{n},v)} \Vert f \Vert _{\dot {M}^{q_{1},\lambda}(\mathbb {R}^{n},v)} \\ &\qquad{} \times \bigl\vert \det A^{-1}(y) \bigr\vert ^{1/q} \bigl\Vert A(y) \bigr\Vert ^{(n+\alpha)(\lambda+1/q)} \bigl\Vert A^{-1}(y) \bigr\Vert ^{\alpha/q}. \end{aligned}$$

Hence,

$$\begin{aligned} \begin{aligned} J_{3}&\preceq v(B)^{\lambda+1/q} \Vert b \Vert _{C\dot {M}O^{q_{2}}(\mathbb {R}^{n}, v)} \Vert f \Vert _{\dot {M}^{q_{1},\lambda}(\mathbb {R}^{n}, v)} \\ &\quad{} \times \int_{\mathbb {R}^{n}}\frac{ \vert \Phi(y) \vert }{ \vert y \vert ^{n}} \bigl\vert \det A^{-1}(y) \bigr\vert ^{1/q} \bigl\Vert A(y) \bigr\Vert ^{(n+\alpha)(\lambda+1/q)} \bigl\Vert A^{-1}(y) \bigr\Vert ^{\alpha/q}\,dy. \end{aligned} \end{aligned}$$

Combining the estimates for $J_{1}$, $J_{2}$, and $J_{3}$ we obtain

$$\begin{aligned} \begin{aligned} \bigl\Vert H_{\Phi,A}^{b}f \bigr\Vert _{\dot {M}^{q,\lambda}(\mathbb {R}^{n}; v)}&\preceq K_{6} \Vert b \Vert _{C\dot{M}O^{q_{2}}(\mathbb {R}^{n}, v)} \Vert f \Vert _{\dot{M}^{q_{1},\lambda}(\mathbb {R}^{n}, v)}. \end{aligned} \end{aligned}$$

This completes the proof of part (i).

(ii) Using Proposition 2.7 along with an argument as given above, the proof of this part becomes simpler. We thus finish the proof of Theorem 4.2.

### Proof of Theorem 4.3

(i) If $\Vert A^{-1}(y) \Vert \preceq \Vert A(y) \Vert ^{-1}$, then (3.7) is valid. The sufficient part of Theorem 4.3 can easily be obtained from Theorem 4.2. Next we will show the necessary part.

For $-1/q<\lambda<0$, choose $f_{0}(x)= \vert x \vert ^{(n+\alpha)\lambda}$. It is easy to see that $f_{0}\in\dot {M}^{q_{1},\lambda}(\mathbb {R}^{n};v)$ and $\Vert f_{0} \Vert _{\dot{M}^{q_{1},\lambda}(\mathbb {R}^{n};v)}= \vert S^{n-1} \vert ^{-\lambda}(n+\alpha )^{\lambda}(1+\lambda q_{1})^{-1/q_{1}}$. Assume that $H_{\phi, A}^{b,1}$ is bounded from $\dot{M}^{q_{1},\lambda }$ to $\dot{M}^{q,\lambda}$ for all $b\in \Vert b \Vert _{C\dot{M}O^{q_{2}}(\mathbb {R}^{n}, v)}$. Taking $b_{0}=\log \vert x \vert $, then by Lemma 2.3 in [31], $b\in C\dot{M}O^{q_{2}}(\mathbb {R}^{n}, v)$. Noting that $(n+\alpha)\lambda<0$, we have

$$ \begin{aligned}[b] H_{\Phi,A}^{b_{0},1}f_{0}(x)&= \int_{ \Vert A(y) \Vert < 1}\frac {\Phi(y)}{ \vert y \vert ^{n}} \bigl\vert A(y)x \bigr\vert ^{(n+\alpha)\lambda}\log \biggl(\frac{ \vert A(y)x \vert }{ \vert x \vert } \biggr)^{-1}\,dy \\ &\succeq f_{0}(x) \int_{ \Vert A(y) \Vert < 1}\frac{\Phi (y)}{ \vert y \vert ^{n}} \bigl\Vert A(y) \bigr\Vert ^{(n+\alpha)\lambda}\log\frac{1}{ \Vert A(y) \Vert }\,dy. \end{aligned} $$

Hence,

$$ \begin{aligned}[b] \bigl\Vert H_{\Phi,A}^{b_{0},1} \bigr\Vert _{\dot{M}^{q_{1},\lambda }(\mathbb {R}^{n}, v)\rightarrow\dot{M}^{q,\lambda}(\mathbb {R}^{n}, v)}& \succeq \int_{ \Vert A(y) \Vert < 1}\frac{\Phi(y)}{ \vert y \vert ^{n}} \bigl\Vert A(y) \bigr\Vert ^{(n+\alpha )\lambda}\log\frac{1}{ \Vert A(y) \Vert }\,dy. \end{aligned} $$

Therefore, we obtain

4.10 $$ \begin{aligned}[b] \int_{ \Vert A(y) \Vert < 1}\frac{\Phi(y)}{ \vert y \vert ^{n}} \bigl\Vert A(y) \bigr\Vert ^{(n+\alpha)\lambda }\log\frac{1}{ \Vert A(y) \Vert }\,dy< \infty. \end{aligned} $$

On the other hand

4.11 $$ \begin{aligned}[b] & \int_{ \Vert A(y) \Vert \le1/2}\frac{\Phi(y)}{ \vert y \vert ^{n}} \bigl\Vert A(y) \bigr\Vert ^{(n+\alpha )\lambda}\,dy \\ &\quad \le \int_{ \Vert A(y) \Vert \le1/2}\frac{\Phi(y)}{ \vert y \vert ^{n}} \bigl\Vert A(y) \bigr\Vert ^{(n+\alpha )\lambda}\log\frac{1}{ \Vert A(y) \Vert }\,dy. \end{aligned} $$

Since $\Phi(y)/ \vert y \vert ^{n}$ is integrable and $(n+\alpha)\lambda<0$,

4.12 $$ \begin{aligned}[b] \int_{1/2\le \Vert A(y) \Vert \le1}\frac{\Phi(y)}{ \vert y \vert ^{n}} \bigl\Vert A(y) \bigr\Vert ^{(n+\alpha )\lambda}\,dy\le\infty. \end{aligned} $$

From (4.11) and (4.12), we have

4.13 $$ \begin{aligned}[b] \int_{ \Vert A(y) \Vert \le1}\frac{\Phi(y)}{ \vert y \vert ^{n}} \bigl\Vert A(y) \bigr\Vert ^{(n+\alpha)\lambda }\,dy< \infty. \end{aligned} $$

It is important to note that

$$ \begin{aligned}[b] K_{8}&=\log2 \int_{ \Vert A(y) \Vert \le1}\frac{\Phi (y)}{ \vert y \vert ^{n}} \bigl\Vert A(y) \bigr\Vert ^{(n+\alpha)\lambda}\,dy \\ &\quad {}+ \int_{ \Vert A(y) \Vert \le1}\frac{\Phi(y)}{ \vert y \vert ^{n}} \bigl\Vert A(y) \bigr\Vert ^{(n+\alpha)\lambda }\log\frac{1}{ \Vert A(y) \Vert }\,dy. \end{aligned} $$

Then, combining (4.10) and (4.13), we have $K_{8}<\infty$.

This proves part (i) of Theorem 4.3.

(ii) In this case we replace $b_{0}(x)$ by $\log\frac{1}{ \vert x \vert }$, then by an argument similar to above the proof can be obtained easily.

## References

[1] Liflyand E, Mórecz F (2000). The Hausdorff operator is bounded on the real Hardy space $H^{1}(\mathbb{R})$. Proc. Am. Math. Soc..

[2] Lerner A, Liflyand E (2007). Multidimensional Hausdorff operators on the real Hardy spaces. J. Aust. Math. Soc..

[3] Andersen K (2003). Boundedness of Hausdorff operators on $L^{p}(\mathbb {R}^{n})$, $H^{1}(\mathbb {R}^{n})$ and $BMO(\mathbb {R}^{n})$. Acta Sci. Math..

[4] Chen J, Fan D, Lin X, Ruan J (2016). The fractional Hausdorff operator on the Hardy space $H^{P}(\mathbb {R}^{n})$. Anal. Math..

[5] Chen J, Fan D, Zhu X (2016). The Hausdorff operator on the Hardy space $H^{1}(\mathbb {R}^{1})$. Acta Math. Hung..

[6] Hung HD, Ky LD, Quang TT (2017). Norm of the Hausdorff operator on the real Hardy space $H^{1}(\mathbb {R})$. Complex Anal. Oper. Theory.

[7] Liflyand E, Miyachi A (2009). Boundedness of the Hausdorff operators in $H^{p}$ spaces, $0 < p < 1 $. Stud. Math..

[8] Lin X, Sun L (2012). Some estimates on the Hausdorff operator. Acta Sci. Math..

[9] Chen J, Fan D, Li J (2012). Hausdorff operators on function spaces. Chin. Ann. Math..

[10] Chen J, Fan D, Wang S (2013). Hausdorff operators on Euclidian spaces. Appl. Math..

[11] Gao G, Zhao F (2015). Sharp weak bounds for Hausdorff operators. Anal. Math..

[12] Ho KP (2016). Hardy inequality and Hausdorff operator on rearrangment Morrey spaces. Publ. Math. (Debr.).

[13] Ho KP (2016). Hardy Littlewood Pólya inequalities and Hausdorff operator on Block spaces. Math. Inequal. Appl..

[14] Liflyand E (2013). Hausdorff operators on Hardy spaces. Eurasian Math. J..

[15] Ruan J, Fan D (2016). Hausdorff operators on the power weighted Hardy spaces. J. Math. Anal. Appl..

[16] Ruan J, Fan D (2017). Hausdorff type operators on the power weighted Hardy spaces $H_{ \vert \cdot \vert ^{\alpha}}^{p}(\mathbb {R}^{n})$. Math. Nachr..

[17] Ruan J, Fan D (2016). Hausdorff operators on weighted Herz-type Hardy spaces. Math. Inequal. Appl..

[18] Ruan J, Fan D, Wu Q (2017). Weighted Herz space estimates for Hausdorff operators on the Heisenberg group. Banach J. Math. Anal..

[19] Gao G, Jia H (2012). Boundedness of commutators of high dimensional Hausdorff operator. J. Funct. Spaces Appl..

[20] Hussain A, Ahmed M (2017). Weak and strong type estimates for the commutators of Hausdorff operator. Math. Inequal. Appl..

[21] Hussain A, Gao G (2013). Multidimensional Hausdorff operators and commutators on Herz-type spaces. J. Inequal. Appl..

[22] Hussain A, Gao G (2014). Some new estimates for the commutators of n-dimensional Hausdorff operator. Appl. Math..

[23] Wu X (2015). Necessary and sufficient conditions for generalized Hausdorff operators and commutators. Ann. Funct. Anal..

[24] Morrey C (1938). On the solutions of quasi-linear elliptic partial differential equations. Trans. Am. Math. Soc..

[25] Alvarez J, Guzmán-Partida M, Lakey J (2000). Spaces of bounded *λ*-central mean oscillation, Morrey spaces, and *λ*-central Carleson measure. Collect. Math..

[26] Lu S, Yang D (1995). The central BMO space and Littlewood operators. Approx. Theory Appl..

[27] Muckenhoupt B (1972). Weighted norm inequalities for the Hardy maximal function. Trans. Am. Math. Soc..

[28] García-Cuerva J, Rubio de Francia J (1985). Weighted Norm Inequalities and Related Topics.

[29] Gundy RF, Wheeden RL (1974). Weighted integral inequalities for nontangential maximal function, Lusin area integral, and Walsh-Paley series. Stud. Math..

[30] Komori Y, Shirai S (2009). Weighted Morrey spaces and a singular integral operator. Math. Nachr..

[31] Chuong NM, Hung HD (2014). Bounds of weighted Hardy-Cesàro operators on weighted Lebesgue and BMO spaces. Integral Transforms Spec. Funct..

